# Tenofovir and Severe Symptomatic Hypophosphatemia

**DOI:** 10.1177/2324709619848796

**Published:** 2019-05-29

**Authors:** Asim Kichloo, Savneek Singh Chugh, Sanjeev Gupta, Jay Panday, Ghazaleh Emedis Goldar

**Affiliations:** 1 St. Mary’s Hospital, Saginaw, MI, USA; 2 Central Michigan University, Mount Pleasant, MI, USA; 3 Westchester Medical Center, Valhalla, NY, USA; 4 Newyork Medical College, Valhalla, NY, USA

**Keywords:** hypophosphatemia, tenofovir, tubular acidosis, Fanconi’s syndrome

## Abstract

Tenofovir is a broadly used drug used for the treatment of human immunodeficiency virus (HIV). Although the initial results of the clinical trials supported the renal safety of Tenofovir, clinical use of it has caused a low, albeit a significant, risk of renal damage either in the form of AKI or CKD. The pathophysiology has been linked to the effect of this medication on the proximal tubular cell. Although the exact mechanism is unknown, studies have suggested that Tenofovir accumulates in proximal tubular cells which are rich in mitochondria. It is both filtered in the glomerulus and actively secreted in the tubules for elimination and is excreted unchanged in the urine. Studies have shown an active transportation of 20-30% of this drug into the renal proximal tubule (PCT) cells via the organic anion transporters in the baso-lateral membrane (primarily hOAT1, and OAT3 to a lesser extent) and ultimate excretion of the drug into the tubular lumen via the transporters in the proximal tubular apical membrane MRP4 and MRP2 (multidrug resistance-associated proteins 2 & 4). Subsequently, the mitochondrial injury caused by Tenofovir can lead to the development of Fanconi’s syndrome which causes renal tubular acidosis, phosphaturia, aminoaciduria, glucosuria with normoglycemia, and tubular proteinuria. Here we present a case where Tenofovir treatment resulted in severe hypophosphatemia requiring hospitalization for parentral phosphate repletion.

## Case Report

A 60-year-old Hispanic female with multiple comorbid conditions including hypertension, type 2 diabetes mellitus, chronic kidney disease stage III with a baseline creatinine of 1.3 mg/dL, baseline chronic obstructive pulmonary disease not on home oxygen, and HIV on highly active antiretroviral therapy (HAART) therapy for more than 10 years, compliant with her medications, visited emergency room with nausea, vomiting, and inability to maintain a good oral intake. She also complained of progressive fatigue over the past several weeks with no relieving factors. Her HAART medications included tenofovir/emtricitabine with fosamprenavir. Her initial workup revealed a serum creatinine of 1.6 mg/dL, phosphorus of 1.4 mg/dL, with rest of her blood work in normal limits. Fractional urinary phosphorus excretion was calculated at 40% despite low phosphorus levels indicating renal loss. Oral phosphate repletion was started; however, tenofovir was continued as per Infectious Disease recommendations. She was subsequently discharged with oral phosphorus supplementation and was advised to follow-up with her primary care physician within 1 week. Before she could follow-up with her primary care physician, she was readmitted with progressive fatigue, loss of appetite, and 1 episode of confusion at home. Workup revealed very low serum phosphorus levels of 0.7 mg/dL. Intravenous phosphorus was initiated for repletion, and after consultation with Nephrology and Infectious Disease specialties, it was decided to stop tenofovir and monitor her serum phosphorus levels. Before discharge, fractional urinary phosphorus excretion showed improvement with a drop to 15%. Her symptoms improved and she was discharged home. Table 1 shows the time course of tenofovir-associated hypophosphatemia in this patient.

**Table 1.** Time Course of Tenofovir-Associated Hypophosphatemia.

	On Presentation	On Readmission	After Tenofovir Discontinuation
Serum phosphorus	1.4 mg/dL	0.7 mg/dL	3.2 mg/dL
Serum creatinine	1.6 mg/dL	1.7 mg/dL	1.4 mg/dL
Fractional excretion of phosphorus	40%	—	15%
Estimated glomerular filtration rate	35 mL/min/1.73 m^2^	35 mL/min/1.73 m^2^	41mL/min/1.73 m^2^

## Discussion

Tenofovir has been a popular choice for use in patients on HAART because of its patient-friendly pharmacodynamics and scheduling. This drug is a nucleotide reverse transcriptase inhibitor, which acts by inhibiting viral RNA directed-DNA polymerase. Studies have shown that tenofovir leads to mitochondrial DNA depletion, especially affecting the mitochondria-rich PCT cells, and ultimately can cause renal cellular injury and development of Fanconi syndrome.^1^ Fanconi’s syndrome is renal proximal tubular dysfunction causing decreased absorption of phosphorous, glucose, and amino acids.^2,3^ This is accompanied by metabolic acidosis secondary to proximal tubular bicarbonate wasting (type II RTA).^4,5^ Most of the cases reported on the kidney injury caused by tenofovir showed some degree of Fanconi syndrome with low or normal glomerular filtration rate.^6-11^ In some patients, the proximal tubulopathy also led to the development of phosphate wasting and/or calcitriol deficiency leading to accelerated bone loss. This is possibly due to the major role of PCT cell’s mitochondria in calcitriol synthesis.^12-15^ In addition, a reduction in glomerular filtration rate and chronic kidney disease-related osteomalacia can have a huge impact on the cardiovascular disease in HIV patients, which is now the leading cause of morbidity and mortality in this population.^16,17^ As mentioned previously, patients on tenofovir usually present with hypophosphatemia, hyperphosphaturia along with glucosuria and aminoaciduria. Significant phosphorous losses in the urine deplete body stores of phosphorous and cause symptomatic hypophosphatemia. Our patient was on tenofovir for HIV and was compliant with her medications. She presented with weakness, nausea, vomiting, and decreased appetite, and on evaluation, she was found to have hypophosphatemia with phosphate levels of 1.4 mg/dL with increased renal excretion of phosphate (urinary fractional excretion of phosphorous was 40%). She was started on oral phosphate repletion and was discharged from the hospital once the levels normalized. However, as a part of her HIV treatment, she was continued on tenofovir, and therefore had an ongoing urinary phosphorous loss despite oral repletion. This led to readmission of this patient with worsening symptoms. Her serum phosphate levels on readmission were 0.7 mg/dL, and she was started on intravenous phosphate repletion to maintain normal serum levels. In an attempt to avoid future recurrence of hypophosphatemia, tenofovir was discontinued. This led to a reduction in urinary phosphate wasting and her urinary phosphate excretion improved to 15%. Patient’s serum phosphate levels normalized and she was ultimately discharged from the hospital.

## Conclusion

It is prudent that health care professionals, especially primary care physicians and Infectious Disease specialists, be aware of the potential side effects of this drug. In addition, physicians must be wary of the possible drug interactions that increase the nephrotoxic profile of tenofovir by favoring its intracellular accumulation, such as nonsteroidal anti-inflammatory drugs and probenecid (both inhibit hOAT1).^18^ Prolonged hypophosphatemia can have a profound effect on multiple organs in the body. It can affect the central nervous system and lead to metabolic encephalopathy due to ATP depletion. This causes a variety of symptoms ranging from mild irritability and paresthesia to more severe symptoms, such as seizures, delirium, and coma.^19-21^ The ATP depletion also affects the cardiopulmonary system by impairing the myocardial contractility.^22^ Studies have shown a higher incidence of ventricular arrhythmias in the setting of acute myocardial infarction in patients suffering from hypophosphatemia.^23^ Therefore, early diagnosis and repletion of phosphorous is of utmost importance.
